# The Role of Journals in Maintaining Data Integrity: Checking of Crystal Structure Data in Acta Crystallographica

**DOI:** 10.6028/jres.101.036

**Published:** 1996

**Authors:** Brian McMahon

**Affiliations:** International Union of Crystallography, 5 Abbey Square, Chester CH1 2HU, England

**Keywords:** crystallographic information file (CIF), data checking, publishing, quality control

## Abstract

Quality control of the papers in its journals is a major concern of the International Union of Crystallography. Recent technological developments, not least the emergence of a standard data interchange file format, have facilitated the checking of numerical data in a paper, and its error-free transference to the printed page. Consequently, database holdings derived from IUCr journals will be of greater accuracy. Other publishers of crystallographic data may benefit from these innovations.

## 1. Setting High Standards

Traditionally, publication of a scientific paper in a peer-reviewed journal has been the recognised manner of reporting a crystallographic structure determination to the scientific community. Since its inception, *Acta Crystallographica*, the flagship journal of the International Union of Crystallography, has placed the highest premium on publishing reliable and accurate structural data. The journal’s Notes for Authors have for many years stipulated detailed criteria for acceptance of a paper reporting a structure determination. An extensive list of requirements endeavoured to ensure that all relevant details of the crystallographic experiment and the interpretation of the collected data were recorded. The policy of requiring the author to supply structure factors, at first for publication in the journal itself, and subsequently (as the sheer volume of experimental data threatened to overwhelm the printed issues) for deposition, was equally designed to allow retrieval and reinterpretation of the information used in solving and refining a structure. This policy has paid dividends in ensuring a consistently high quality of published structures, and in permitting the re-evaluation and subsequent correction of a number of incorrect structure determinations (see for instance Marsh and Herbstein [1]).

As x-ray crystallography has evolved from being a novel and difficult technique into the routine (and often semi-automatic) everyday tool of the modern structural chemist and solid-state scientist, so has there been a large growth in the number of structures reported in brief in the journals. *Acta Crystallographica* still publishes many seminal papers in structural science, where the structures reported yield fresh insights into the nature and chemistry of the materials described. But at the same time, very many structures are described which have no more profound impact than their own inherent interest, and a large collection of these is found in *Section C* of the journal (itself a descendant of the short *Crystal Structure Communications* published by the University of Parma in the years 1972–1982). In the last few years, the IUCr has devoted a significant amount of effort to developing its publication procedures for *Section C* in a manner that is novel, efficient, and yet preserves—indeed, enhances—the rigour of its quality control. Many of the techniques described below apply also to the other sections of the journal, wherever structural reports are published; but it is in *Section C* that they have been most highly developed.

## 2. The Crystallographic Information File

Many of the recent developments in the production of *Acta Crystallographica* have arisen as a direct consequence of the adoption by the IUCr of the Crystallographic Information File (CIF) [2] as the standard file format for crystallographic information interchange. The original mandate for the development of such an interchange file format allowed for it to act as a medium for publication, but only through the mechanism of an embedded text field intended to carry the complete contents of the submitted paper. Such a mechanism had already been incorporated in an earlier such initiative, the Standard Crystallographic File Structure [3], but for a variety of technical reasons this had never enjoyed much success, and no papers were ever published in Acta through this mechanism.

However, it was realised that a CIF could be used for publication in a much more powerful way. The Commission on Journals requirements for a published structure (as listed in regular Notes for Authors in the journal) formed an extensive list of well-defined quantities that would normally be written by standard software into designated fields in a CIF. It would clearly be possible to extract this information automatically from the file, rearrange and format it in a way suitable for publication, and thereby construct the bulk of the numerical and experimental data normally collected and published in an *Acta* paper.

The discursive text of the paper (especially in the case of short reports as typically published in *Section C*) could easily be added to the file as a number of brief textual fields. According to this philosophy, the mundane task of assembling the account of the experimental conditions, and of collating the final calculated atomic coordinates and derived geometry lists, could be removed completely from the author and left to the software packages with which he or she was already working.

The promise of this approach was so appealing that the IUCr encouraged submission of papers in CIF format to *Section C* of *Acta Crystallographica* as soon as the CIF specification was published, and a trickle of such machine-readable submissions began arriving at the *Acta* editorial offices within a few weeks of the call for such papers.

Initially, however, very little software existed that was capable of writing files of the new format, and the first submissions were often constructed by hand using text editors or simple scripts and macros devised by authors. It is an eloquent testimony to the simplicity of the file structure that this could be done at all; but, at the same time, there were sufficient unfamiliarities and subtleties of the new type of file to occasion several authors some trouble in constructing a satisfactory submission.

**Fig. 1:**
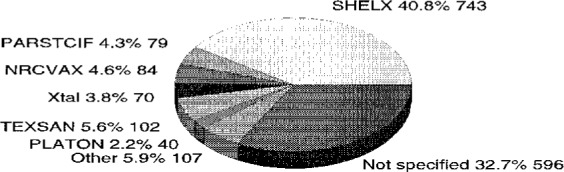
Proportion of CIF submissions generated by various software packages as held in Chester at the end of 1995. In addition to the 1821 files represented here, 3320 CIFs had been created by inhouse software at Chester.

It was only with the development of additional CIF writing software during the next few years that CIF submission became a routine technique for the crystallographic author. Probably the most significant event in encouraging widespread adoption of CIF submission was the issue in 1993 of a greatly improved version of the well known refinement program SHELXL-93^1^ [4], though the availability of CIF generators in packages such as Xtal [5] and NRCVAX [6] was also important for users of larger integrated packages of crystallographic software. Figure 1 illustrates the variety of software used by authors submitting CIFs to *Acta C*.

**Fig. 2:**
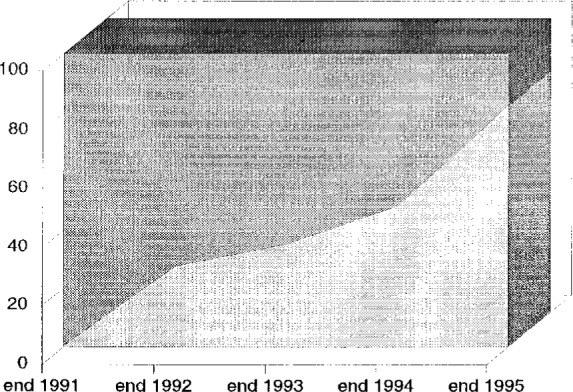
Growth in percentage of *Acta C* submissions made in CIF format from end of 1991 to end of 1995.

From slow beginnings at the start of 1992, CIF submission to *Section C* increased gradually until a large majority of papers reached the journal in the approved format by late 1995. CIF submission will be the sole recommended method of submitting a paper to *Section C* from January 1996. The growth in the number of electronic submissions is displayed in Fig. 2.

An important factor in encouraging this shift in the way authors submitted their papers was the strenuous effort put into educational activities by the journal. From the beginning, *Acta* staff corrected files that were syntactically incorrect, and returned the modified files to the submitting authors, often with detailed explanations of the corrections that had been made. Copies of CIF manipulation software, such as the field getter and manipulator QUASAR [7] and the data name checker CYCLOPS [8], were sent by email to contributing authors. Informal articles explaining the use of the new file appeared in *Acta Crystallographica* [9] and in other publications. Tutorials and workshops were held at crystallographic meetings. Notes for Authors were continually revised to clarify the use of CIF, and a booklet aimed directly at authors [10] was produced and distributed. Automatic network services, to be described below, were introduced to allow authors to understand better how their files were handled.

While these activities undoubtedly required a great investment of time and effort, the gains were seen to be well worthwhile. *Acta C* is typeset entirely from CIFs (even the few remaining hard-copy submissions are encoded in CIF format at the editorial offices). The CIFs, subjected prior to acceptance to a very wide range of checks, are suitable for direct deposition into major databases. Further, the files stored at the editorial office are suitable for manipulation into various different forms and formats, and so are an ideal resource for electronic publication using a variety of existing and evolving techniques. The checking and formatting software developed for the first tentative submissions has been able to handle effortlessly CIFs generated by a wide range of originating software packages, and has needed only to be enhanced, never rewritten.

The change in working practices in the editorial office has been immense; the changes required of the authors have been substantial; and the change in the role of the journal is likely to prove profound.

## 3. Checking of Structural Papers

Another element of the revolution in handling structural data in the journal has been the consistent and detailed checking of the numerical information in submitted papers. It was long considered an essential part of the refereeing process for *Acta* papers that the correctness of the numbers in the paper should be checked wherever possible. Traditionally, co-editors scrutinised and analysed the numerical content of the papers they received. The intention was to catch major errors of interpretation, or problems such as the interpolation of a set of data from a different result set; but also the conscientious co-editor was aware that random keyboarding errors might have been introduced by the author, or the author’s secretary, and no effort was spared to try to detect such minor errors. However, even the detection of such errors could not guarantee the absolute quality of the printed paper—further keyboarding errors could always be introduced in the typesetting and proof correction stages of production.

Although this system worked well for many years, and ensured a high level of accuracy, it placed a large burden of work on co-editors who had to enter the data for checking into files suitable for input to the checking software they were using; and there was a great deal of variation between co-editors in their ability and resources to undertake this work. Consequently, it was decided in the mid-1980s to transfer progressively the checking function from the co-editors to the editorial staff. This would ensure that ultimately all papers would be subjected to a rigorous and consistent checking procedure, and it would allow the editorial staff to accumulate a knowledge of structure checking second to none.

The enterprise was assisted by the generous donation by a number of software authors of their checking programs, so that the editorial office was able to utilize a greater range of programs than would have been available to any individual co-editor.

Trials began in 1989, when papers handled by a number of co-editors were systematically checked for internal consistency and for the reasonableness of their space group assignments. It was clear that the burden of entering the data for checking would impact heavily on the editorial process, but the longer-term benefits of following through such a consistent checking policy were regarded as sufficient to justify further effort. During the early trials, work on defining the new Crystallographic Information File was being conducted by a Working Party on Crystallographic Information established by the IUCr Commissions on Data and Journals. It seemed appropriate to adopt this emerging standard as the in-house means of storing the data entered for numerical checking. Data could be extracted as required from the CIF, and reformatted for input according to the needs of the various programs used. While this choice was entirely for working convenience, it clearly was of immense benefit for the handling of papers that would in the future be submitted in CIF format. It is again noteworthy that the software developed at that time for translating CIF data into the large number of different file formats needed by the checking programs is still in use for all current submissions.

The checking procedures currently concentrate on two main aspects: the internal consistency of the geometric model reported by the author; and the reasonableness of the symmetry description of the structure.

The most useful program for checking the geometry is the UNIMOL package developed by the Cambridge Crystallographic Data Centre for just this purpose [11]. This software takes the given atomic coordinate set and space group, and builds a connected model of the structure, transforming coordinates with the symmetry operations of the space group as necessary to build the most compact residue or set of residues comprising the asymmetric unit. All bond distances are then calculated and compared against an input set of bond distances supplied by the author. Major discrepancies in the distances or their standard uncertainty values (s.u.’s, or e.s.d.’s as they were formerly known) are flagged in the output file. The software will also perturb the positions of individual atom sites participating in mis-matched bonds to seek for a more consistent set of atomic positions. If a very large proportion of bonds are apparently in error, the cell constants will be varied in an effort to restore some reasonableness to the reported structure. It is often the case that the suggested coordinates (or cell parameters) resulting from these calculations are seen to differ from those reported by the interchange of a pair of digits—a common keyboarding error.

The original UNIMOL package has been modified and redesigned for use by the CCDC staff, and in the spirit of continuing cooperation between the CCDC and *Acta Crystallographica*, the new software packages BUILDER [12] and PREQUEST [13] are also used by the *Acta* checking staff. The functionality of the original software remains, but the new packages allow structures to be analysed interactively within an X11 graphics environment, and are far better for visualising disorder and polymeric structures than the original program. The ability to expand the structure around any arbitrary origin makes them better suited for investigating non-molecular structures than the venerable UNIMOL program.

Because of their facility for automatic comparison, the Cambridge programs allow for a very rapid evaluation of the consistency of a molecular model. Nonetheless, an author will often describe other aspects of intra- or intermolecular geometry, and other programs are used to check the reported values of angles, torsion angles, best least-squares line and plane parameters, and other features. Among the most comprehensive are the PARST library of routines [14] originally used in checking papers submitted to *Crystal Structure Communications*; and PLATON [15], a very comprehensive program package which includes, amongst its other features, the ability to populate a cell volume on the basis of the reported atomic coordinates and chemical types, and search for residual solvent-accessible voids of sufficient size to accommodate solvent molecules which have been left out of the refinement.

Numerous other programs are used to check geometry elements, including the DISPOW routine of NRC-VAX [6] and the BONDLA module of Xtal [5]. While many of these generate essentially the same results, it is often useful to be able to search for some feature that is more easily found in the layout of one program as opposed to another. Occasionally, different programs will use different conventions (for example in the choice of orthogonal coordinate axes) and a checking run using the author’s conventions can be quicker than calculating or applying the relevant transformation. And it is useful to be able to compare the results of different program packages across a large collection of input data sets; though to my knowledge no genuine bugs have yet been thrown up in any standard package as a result of this approach!

Most of the programs listed run in a batch mode, taking the input data for one (or more) structures and producing extensive listings of all derivable values. Increasingly, however, it is convenient to run interactive graphics programs that allow the user to examine and explore different parts of the structure with point-and-click mouse techniques. The adoption of this methodology by the BUILDER and PREQUEST programs has already been mentioned. Other programs, such as the portable interactive graphics (PIG) module of Xtal [5], the graphics program PLUTON [15], and some of the graphics subroutines of NRCVAX [6] are routinely used in this way.

Most of the software in use for structure checking is easiest to use with molecular structures, though programs such as STRUMO [16], developed specifically for inorganic modelling, are also available. Nevertheless, it remains true that the effective visualisation and description of inorganic structures, especially of high symmetry, poses a challenge to the existing checking software.

The other major concern in checking is the reasonable assignment of a space group to the structure reported. A number of cell reduction programs are available to check on the metric symmetry of a cell lattice, including DELOS [17], TRACER [18] and CREDUC [19]. Other approaches are also taken, such as that of NEWLAT [20], which generates a set of new lattices from the metric tensor constructed from an input lattice, and assigns to each new lattice an empirical figure of merit; and the converse-transformation algorithm of NIST*LATTICE [21].

Two more powerful programs explore the symmetry of the occupied atomic positions. MISSYM [22] generates all possible symmetries for a lattice deduced from the cell reduction algorithm CREDUC; and then applies these to each atomic site, searching for coincident transformed sites which must arise from higher symmetry than implied by the space group reported.

BUNYIP [23] also searches for extra symmetry elements between the reported atomic positions, this time by constructing all interatomic vectors between members of the asymmetric unit and analyzing the locus of mid-points of these vectors. Where the locus is a well defined geometric object, such as a point or a line, additional symmetry elements must be present.

Both MISSYM and BUNYIP may indicate pseudosymmetries or symmetry elements relating parts of a structure, and so suspect features they may report are not invariably evidence of error; nonetheless, it is generally the case that any features they do reveal are of sufficient interest to merit a discussion in the paper.

Although there will always be subtle cases where the correct symmetry of a crystal structure cannot be unequivocally determined, it is nonetheless true that many of the structures that have been flagged as suspect by these programs have been refined in a different space group prior to publication; and the number of erroneous space groups reported in *Acta Crystallographica* appears genuinely to be on the decline.

## 4. Methodology of Editorial Checking

An important feature of the implementation of the checking software at the *Acta* offices is that, although very few changes have been made to the programs, they are run in a homogeneous and flexible operating system environment that allows them to be used with maximum flexibility. Several of the programs were written as batch programs, designed to process dozens of structures in long uninterrupted runs. However, we run each program on a single structure at a time, and have designed the operating environment to allow rapid interaction with the program and its result files, enhancing the ability of the editorial staff to interact and experiment with the structure they are analyzing.

Each paper is managed as a single CIF that may contain several structures (each occupying a separate data block within the file). For each structure a separate directory is created, and the directory is populated with the input files for checking programs appropriate to that structure. Each file has an associated icon in the visual file manager that the operating system supports (typically SunOS version 4.1.3 with the Sun OpenWindows window manager on a SPARC workstation). This is true of both input and output files. Double-clicking on an icon invokes an associated application. In the case of output files, this is simply a matter of opening the file in a scrollable text editor, though the width of the open window is tailored to the width of the output listing. For input files, activating the relevant icon runs the checking program associated with that icon.

Hence, the normal method of checking the contents of a file is to double-click on the CIF icon associated with a specific paper. Subdirectories are automatically created, one per structure, and a set of standard checking programs is run (with default parameters) for each structure. A summary of results is written to the screen as the checks progress. When the batch of checks is complete, the checking staff member may choose any of the sub-directories and examine in more detail the output from any of the many programs run.

On occasion, it may be necessary to rerun an individual program—the translation of a particular data field was not correct, for example, or the program should be run with nonstandard values for some of its parameters in this instance. It is simply a case of editing the input file, saving the edited changes, and double-clicking on the icon associated with that file to re-run that specific program.

It may be that one or several of the checking programs have indicated an error in the structure. In that case, the icon representing the CIF in the current subdirectory is selected to invoke a text-editing tool, and the relevant portion of the file changed. (It should be explained that this icon is a symbolic link to the file in the parent directory.) When all suitable changes have been made, the CIF icon is double-clicked, and the entire set of checks is re-run for the current structure.

If more far-reaching changes are involved, the copy of the CIF in the parent directory is edited, its icon double-clicked, and all checks are re-run on all structures described in the file.

This environment affords maximum flexibility to the checking staff in their handling of CIFs and the various structures that may be described in a single CIF. It has proven popular among the editorial staff, and has greatly facilitated the efficient and rapid processing of large numbers of structures to be checked.

Certain other checks are also carried out on each new submission, to search for prior publication or attempted resubmission of a rejected paper. The literature checks include an automatic search of the NIST Crystal Data one-line database [24], based on reduced-cell volumes; and a formula check against the Inorganic [25] and Cambridge Structural [26] databases. This latter is still carried out manually by editorial staff, using the inverted files built and maintained within the Daresbury Laboratory Crystallographic Databases System [27].

## 5. Automated Typesetting

Although CIF was never designed as a page-formatting system, it offers great benefit to publishers in its clear tagging of specific items of information. Such detailed structure plays the same role as the generalised markup system developed for electronic publication files as the ISO Standard SGML [28]. In the case of *Acta C*, the format for structural papers was always well defined, and it proved very simple to transform the data stored in a CIF to the printed page.

The techniques employed are very straightforward. An input CIF is passed through a filter which reorders the data items it contains to conform to the requirements of the printed paper (it is a design element of CIF that specific data may be located wherever convenient in the file, whereas the order of presentation in the paper must conform to editorial house rules), and then translates the file into an input format used by the publicly available TeX typesetting system [29]. Each data name listed in the CIF Core Dictionary [2] is associated through a map file with a macro in the TeX language, and the value of the data is passed as an argument to the TeX macro. Hence the list of cell parameters in a CIF, e.g.


_cell_length_a10.452(3)_cell_length_b11.664(4)_cell_length_c15.641(4)_cell_angle_alpha94.37(2)_cell_angle_beta89.75(2)_cell_angle_gamma111.87(2)_cell_volume1763.8(8)is translated simply to the list

\cella{10.452 (3)}
\cellb{11.664 (4)}
\cellc{15.641 (4)}
\cellalpha{94.37 (2)}
\cellbeta{89.75 (2)}
\cellgamma{111.87 (2)}
\cellvol{1763.8 (8)}
where each macro is defined within another control file to format its argument. Thus, the definition for \cella instructs the formatting program to typeset on a fresh line an italic letter a, followed by an equals sign, then the argument of the macro, then a space and the symbol for an ångström unit. Hence the example block is typeset as

*a* = 10.452 (3) Å
*b* = 11.664 (4) Å
*c* = 15.641 (4) Å
*α* = 94.37 (2) °
*β* = 89.75 (2) °
*γ*= 111.87 (2) °
*V* = 1763.8 (8) Å^3^

Longer blocks of continuous prose are handled in an analogous way: again, they are passed as the argument to a typesetting macro, but in this case the macro contains detailed instructions about typefaces, typesizes, justification and spacing. A small set of simple codes to represent Greek and some mathematical symbols is available to the author for incorporation into the text.

Tables are also built from the looped data structures in CIFs. The most complex tables sometimes found in printed papers are not easily handled by this approach, but it is rare for the standard papers published in *Section C* to require these, and work is in hand to explore ways of representing tabular material in other publications. The difficulty of handling complex tables has less to do with the development of formatting instructions than with the desire not to burden the contributing author with the need to specify typographic formatting. Except for knowledge of the few codes for special characters, the author is freed entirely from concerns over the layout of the finished paper. As might be expected, earlier authors sometimes found this troubling, but gradually people are beginning to realise and enjoy the liberating influence of not having to worry about journal style. Since the author’s refinement program supplies the bulk of the contents of a CIF, and the author need only add some paragraphs of explanatory text, much of the laborious business of preparing the paper for publication has been simplified.

Because the TeX macros are defined in an external control file, it is simple to exchange one set of definitions for another; and so the CIF, when received at the editorial office, is cast into a format convenient for inspection and annotation by a referee—in a large typeface, double-spaced and set on a wide margin. When the file (possibly corrected) is finally ready for publication, another pass through the formatter with a different set of macro definitions generates a proof in the style of the journal. It may at once be recognised that the ability to typeset an entire paper in a few seconds from the submitted CIF has significant implications for the economics of the typesetting process.

More valuable to the crystallographic enterprise, however, is the fact that the data travel from the refinement package to the printed page without the need for any manual keyboarding, and so the simple typographic errors that have always proved so difficult to guard against in conventional publishing are entirely eliminated. It is, of course, possible that errors may be introduced whenever the file is edited, for whatever reason; but the speed and convenience of the checking procedures mean that it is always possible to recheck swiftly any file suspected of error. In practice, not every possible check will always be carried out; but it is nevertheless the case that this approach to publishing will secure a much lower error rate in the final product.

## 6. Other Applications of Automated Typesetting from CIF

It has already been pointed out that the translation from an input CIF to a TeX file suitable for printing as a journal article succeeds in the case of an *Acta C* paper because the short structure reports themselves have a very well defined layout and content. It is not expected that this approach can be applied so thoroughly across all the IUCr journals. Nevertheless, some of the other publishing activities of the Union have been able to make use of this technique. Two are briefly discussed here.

The dictionary of universally recognised data names for CIF is itself a file in CIF-like format, where the definitions and attributes of data names are stored in a file that can be manipulated by standard CIF software. It is therefore straightforward to typeset the printed form of the dictionary in the same way as *Section C* papers are produced, and the resultant print dictionary is fully consistent in its internal style. More importantly, the data names defined within the dictionary can be checked by software for consistency and accuracy in cross-referencing, and the resulting checked data names are transferred to print without re-keyboarding and consequent introduction of typographic errors—another example of the benefits of electronic checking.

This ease of production of a typeset dictionary has been invaluable during the protracted development of the mmCIF dictionary for macromolecular crystallography [30], when frequent revisions of a document in excess of 100 pages needed to be produced rapidly (and cheaply) for a small group of expert reviewers.

The second such application was the production of the Ninth Edition of the *World Directory of Crystallographers* [31], again from a set of files, this time of biographical data, in a CIF-like format. While previous editions of this directory had explored different approaches to computerized production, the length of time taken to collect the published data, and the long and often complex printing processes involved, usually doomed the directory to obsolescence even before it was completed. On this occasion, the data were collected in the familiar CIF-like format, though again the collection of 8000 entries from all over the world took a longer time than was desirable. However, it was then simple to format each entry as a separate proof sheet to be sent directly to the person described; errors and alterations were emailed or faxed to the Union editorial offices, and a highly accurate and up-to-date printed edition was produced within a few weeks.

The structure of the *World Directory* entries again allowed for a certain amount of dynamic checking of contents. For instance, the interests field could be checked automatically against a list of approved keywords.

Furthermore, the directory contents were also easily translated into a database format suitable for online interrogation by Internet users. This was a simple, but effective, demonstration of the ready interconvertibility of different forms of a well defined data set—a lesson that has not been overlooked in considering the future nature of the IUCr journals themselves.

## 7. Shifting the Burden of Checking Towards the Author

Although the checking procedures instituted at the IUCr editorial offices have had a large beneficial effect on the quality of published papers, they are quite labour-intensive to implement. Ideally, of course, the author would adopt full responsibility for the accuracy of his or her reported data, and the IUCr checks would be able to confirm that accuracy routinely and automatically. However, there are subtle experimental errors that can creep into crystallographic results, and not all authors possess appropriate software (or, in some cases, experience) to check for all of these. In recognition of this, the IUCr has provided a simple interface to its checking software for the use of prospective authors.

The author may send a copy of his or her data, in CIF format, to an electronic mail address (check-cif@iucr.ac.uk). The CIF is checked for syntactic correctness, and the numerical data are checked using some of the programs routinely employed for the full checking of submitted papers. A summary report of any errors or anomalies is mailed back to the author.

The checking software used in this process must produce clear and concise diagnostic reports, and so it has not yet proven possible to apply the complete range of checks available to the checking staff. Nevertheless, as more software is fine-tuned to operate smoothly within this procedure, it is expected that progressively more detailed reports will be generated and returned to authors. Already the service is in regular use by intending authors, and provides an important check on the crystal cell parameters and assigned space group.

Although the service is provided for the benefits of authors intending to submit papers to IUCr journals, no limitations are imposed on the identity of users or frequency of use, so that authors intending to submit papers to other journals, or indeed researchers wishing to check the validity of their own results (not intended for publication) may freely make use of this facility. It is hoped that this informal usage will raise the standards of crystal structure reports throughout the community.

It is, of course, quite possible that other journals may wish to apply such checks to the crystal structure data that they report, and the IUCr is interested in the possibility of providing such a service to other publishers of crystallographic information. The objective of improving the overall standard of reported structures (at modest cost) is one that should appeal to all serious publishers.

There is another potential benefit of using CIFs as the standard transfer mechanism for data. The required information content of a submission to *Acta Crystallographica* is specified by a list of data items that should be present in the CIF. In like manner, other journals may specify their requirements by supplying a list of required data names. One may envisage the emergence of a base set of required data names common to all journals, so that crystallographic material submitted even as a file for deposit with a chemistry journal, for instance, would be guaranteed to possess at least some minimum content.

## 8. Printcif

The IUCr also provides an email-based facility (printcif@iucr.ac.uk) for formatting CIFs in an attractively typeset style. This is also provided as a service to intending authors, to demonstrate the way in which their data file will be transformed into print. It provides a preprint that may, for example, be supplied to employers who examine their employees’ work prior to submission to journals.

Although the preprint generated by this service is in a style appropriate to the IUCr journals, it is quite feasible to change the typographic style to suit the requirements of individual submitters, and this is an application with potential benefit to users requiring a particular house style for representing crystal structure information in print.

## Footnotes

1 Certain commercial equipment, instruments or materials are identified in this paper to foster understanding. Such identification does not imply recommendation or endorsement by the National Institute of Standards and Technology, nor does it imply that the materials or equipment identified are necessarily the best available for the purpose.
